# Evaluation of the impact of systemic dexamethasone dosage on docetaxel-induced hand-foot syndrome in patients with breast cancer

**DOI:** 10.1038/s41598-024-64553-z

**Published:** 2024-06-18

**Authors:** Yoshitaka Saito, Yoh Takekuma, Masato Takahashi, Tomohiro Oshino, Mitsuru Sugawara

**Affiliations:** 1 Department of Clinical Pharmaceutics & Therapeutics, Faculty of Pharmaceutical Sciences, Hokkaido University of Science, 4-1, Maeda 7-jo 15-chome, Teine-ku, Sapporo, Hokkaido 006-8585 Japan; 2 Department of Pharmacy, Hokkaido University Hospital, Kita 14-jo, Nishi 5-chome, Kita-ku, Sapporo, 060-8648 Japan; 3 Department of Breast Surgery, Hokkaido University Hospital, Kita 14-jo, Nishi 5-chome, Kita-ku, Sapporo, 060-8648 Japan; 4 Laboratory of Pharmacokinetics, Faculty of Pharmaceutical Sciences, Hokkaido University, Kita 12-Jo, Nishi 6-chome, Kita-ku, Sapporo, 060-0812 Japan

**Keywords:** Hand-foot syndrome, Dexamethasone, Docetaxel, Dose-dependent, Breast cancer, Cancer, Breast cancer

## Abstract

Hand-foot syndrome (HFS) is a frequently occurring and treatment-requiring adverse effect of docetaxel. We previously reported that systemic dexamethasone (DEX) prevents the other docetaxel-induced adverse inflammatory effects in a dose-dependent manner. This study aimed to evaluate the dose-dependent efficacy of systemic DEX in attenuating HFS in patients with breast cancer receiving docetaxel. Patients with breast cancer receiving docetaxel (75 mg/m^2^)-containing regimens (n = 111) were divided into 4 and 8 mg/day DEX groups, with each DEX dose administered on days 2–4, and analyzed retrospectively. Development of all-grade HFS in all treatment cycles was significantly lower in the 8 mg group (50.0%) than in the 4 mg group (73.0%, *P* = 0.03), with primary endpoint accomplishment. Moreover, its development in the first cycle was also lower in the 8 mg group than in the 4 mg group. These results were confirmed in a propensity score-matched population. Logistic regression analysis suggested higher DEX dosage as an independent preventive factor (adjusted odds ratio 0.35; 95% confidence interval 0.14–0.86, *P* = 0.02 for all cycles; 0.26, 0.11–0.63, *P* = 0.003 for the first cycle). Our study suggests that systemic DEX prevents the occurrence of docetaxel-induced HFS in patients with breast cancer in a dose-dependent manner in a real-world setting.

## Introduction

The prevalence of breast cancer is increasing worldwide, but considerable improvements have been made in its detection and treatment^1^, which has led to a reduction in the associated mortality rate^1^. Chemotherapy, in particular, plays an important role in the treatment of breast cancer in perioperative and metastatic settings^1^. As chemotherapy induces adverse effects resulting in a decrease in the quality of life (QOL) of patients, its management is essential for providing quality treatment.

Docetaxel is a key drug used in the management of breast cancer^2–4^. However, its administration causes adverse effects such as severe neutropenia, fluid retention, peripheral neuropathy, pain, oral mucositis, and skin toxicities^2–4^. Hand-foot syndrome (HFS) is one of the most frequently appearing and treatment-requiring symptoms of docetaxel administration. HFS is initially characterized by palmoplantar numbness, tingling, or burning pain with sharply demarcated erythema, with or without edema, cracking, or desquamation^5^. Factors reportedly associated with development of HFS are female sex; good performance status (PS); high dose intensity; high tumor- and human epidermal growth factor receptor 2 (HER2)-positive percentages; high body surface area (BSA) or body mass index (BMI); low albumin level, high transaminase level, and high white blood cell count at baseline; concomitant use of renin-angiotensin system inhibitors (RASIs) and bevacizumab; history of fluorinated pyrimidine administration; complication of diabetes mellitus (DM); pre-existing inflammatory disease; presence of lung and liver metastases; number of affected organs; warm hands, good peripheral blood circulation, and excessive sweat excretion; and history of gallstones^6–11^.

Docetaxel-induced HFS reportedly occurs in 6–58% of patients who receive the drug. This statistic includes 0–4% of patients with severe manifestations of HFS^12–15^. Severe symptoms can induce dose reduction, suspension, or discontinuation of the treatment^12,14,16^. However, skin toxicities are commonly under-reported and not systematically ascertained^16^. Inflammation is one of the most common causes of HFS^5,12^, and topical steroid application is the standard treatment for typical HFS presentation^12^. On the other hand, dexamethasone (DEX) is generally co-administered with docetaxel for several days to prevent fluid retention. However, its suggested dosages and administration durations vary (8–16 mg/day for 3–5 days)^3,4,17–20^. At our facility, the initial dose of DEX administered was 4 mg orally on days 2–4, which was increased to 8 mg daily in July 2017 in accordance with the aforementioned reports^3,17,19^. We evaluated the impact of the change in systemic DEX dosage on other inflammatory adverse effects, such as taxane-associated acute pain syndrome (T-APS) and oral mucositis, and found that increase in DEX dose resulted in attenuation of the symptoms^21,22^. No previous study has evaluated the influence of systemic corticosteroids on HFS. If this change also ameliorates HFS secondarily, it may be a promising prophylaxis.

In the present study, we aimed to evaluate the dose-dependent efficacy of systemic DEX in attenuating HFS in patients with breast cancer receiving docetaxel-containing treatment in a real-world setting.

## Results

### Patient characteristics

**Figure 1:**
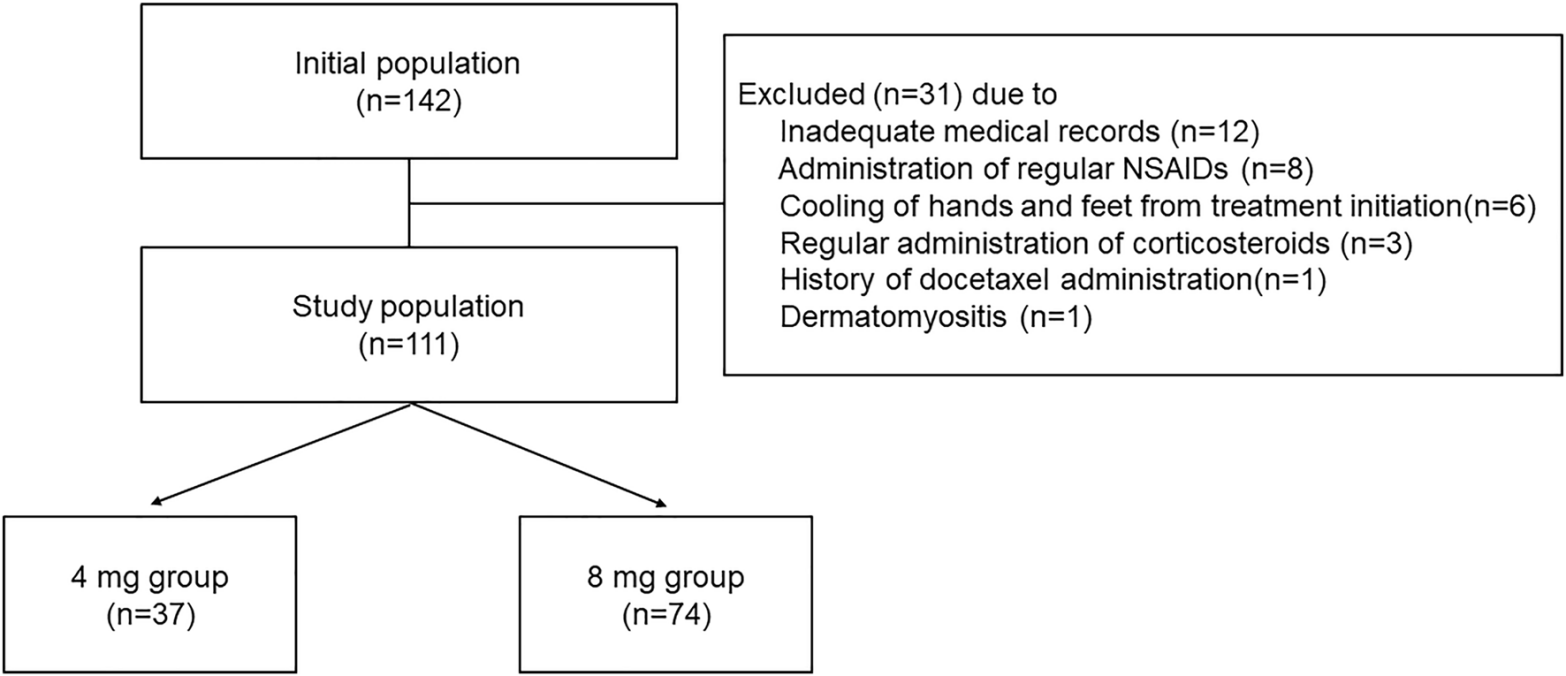
Design of this study. *NSAIDs* non-steroidal anti-inflammatory drugs.

A total of 111 eligible patients were enrolled in this retrospective observational study (Fig. 1). The baseline patient characteristics are described in Table 1. In the all-patient population, there were no significant differences between the two groups in terms of Eastern Cooperative Oncology Group performance status (ECOG-PS), staging, hormonal receptor expression, HER2 overexpression, Ki-67, existence of lung or liver metastasis, BSA, BMI, neutrophil count, hemoglobin level, platelet count, liver dysfunction (grade 1 or higher aspartate aminotransferase, alanine aminotransferase, and total bilirubin level elevation), regular alcohol intake (≥ 5 days in a week), smoking history, complications of DM, prior treatment history, and co-administration of pegfilgrastim and RASIs. However, patients in the 8 mg group were significantly older and had lower creatinine clearance (CCr) and serum albumin levels, with significantly more pertuzumab co-administration. In contrast, no background differences were observed in the propensity score-matched population.

**Table 1:** Patient characteristics.

	All-patient population			Propensity score-matched population		
	4 mg group (n = 37)	8 mg group (n = 74)	*P* value	4 mg group (n = 31)	8 mg group (n = 31)	*P* value
Age (median, range)	51 (30–63)	56 (27–73)	0.004**	52 (34–63)	50 (27–73)	0.99
Performance status (ECOG) (n, %)						
0–1	37 (100%)	74 (100%)	1.00	31 (100%)	31 (100%)	1.00
Staging (n, %)						
I–III	35 (94.6%)	64 (86.5%)		30 (96.8%)	31 (100%)	
IV/recurrence	2 (5.4%)	10 (13.5%)	0.33	1 (3.2%)	0 (0%)	1.00
Hormonal receptors (n, %)						
ER, PR-positive or both	20 (54.1%)	40 (54.1%)	1.00	15 (48.4%)	19 (61.3%)	0.44
HER2 overexpression (n, %)	12 (32.4%)	28 (37.8%)	0.68	10 (32.3%)	5 (16.1%)	0.24
Ki-67 (%) (median, range)	50.4 (1.3–97.6)	32.8 (1.6–95.9)	0.16	50.1 (1.3–97.6)	35.1 (7.3–95.9)	0.18
Presence of lung metastasis (n, %)	2 (5.4%)	4 (5.4%)	1.00	1 (3.2%)	0 (0%)	1.00
Presence of liver metastasis (n, %)	0 (0%)	2 (2.7%)	0.55	0 (0%)	0 (0%)	–
BSA (m^2^) (median, range)	1.53 (1.40–1.94)	1.55 (1.30–2.00)	0.93	1.56 (1.40–1.94)	1.59 (1.34–2.00)	0.79
BMI (kg/m^2^) (median, range)	20.7 (17.9–36.5)	23.2 (16.3–38.3)	0.08	20.7 (18.5–36.5)	23.2 (17.1–36.6)	0.25
Neutrophil (/μL) (median, range)	2895 (910–9944)	3134 (1007–17,020)	0.14	2622 (910–9944)	2809 (1372–6188)	0.35
Hemoglobin (g/dL) (median, range)	11.9 (9.4–15.4)	11.4 (9.1–14.5)	0.05	11.7 (9.4–15.4)	11.8 (9.4–14.5)	0.86
Platelet (× 10^3^/μL) (median, range)	265 (139–413)	283 (93–618)	0.27	261 (139–404)	296 (93–430)	0.07
Liver dysfunction (n, %)	21 (56.8%)	35 (47.3%)	0.42	14 (45.2%)	14 (45.2%)	1.00
CCr (mL/min) (median, range)	99.9 (74.3–211.4)	89.4 (60.2–234.5)	0.002**	105.2 (74.3–211.4)	94.2 (65.3–234.5)	0.40
Serum albumin (g/dL) (median, range)	4.1 (3.7–5.0)	4.0 (3.2–4.9)	0.008**	4.1 (3.7–5.0)	4.1 (3.6–4.9)	0.51
Alcohol intake (≥ 5 days in a week) (n, %)	8 (21.6%)	9 (12.2%)	0.26	6 (19.4%)	5 (16.1%)	1.00
Smoking history (former or current) (n, %)	21 (56.8%)	35 (47.3%)	0.42	18 (58.1%)	14 (45.2%)	0.45
Current smoker	5 (13.5%)	12 (16.2%)	0.79	3 (9.7%)	1 (3.2%)	0.61
Complications of diabetes mellitus (n, %)	1 (2.7%)	6 (8.1%)	0.42	1 (3.2%)	2 (6.5%)	1.00
Prior treatment history (n, %)	27 (73.0%)	57 (77.0%)	0.65	24 (77.4%)	25 (80.7%)	1.00
Co-administration of pertuzumab (n, %)	1 (2.7%)	22 (29.7%)	0.0005**	1 (3.2%)	0 (0%)	1.00
Co-administration of pegfilgrastim (n, %)	3 (8.3%)	13 (17.6%)	0.25	3 (9.7%)	3 (9.7%)	1.00
Co-administration of RASIs (n, %)	2 (5.4%)	7 (9.5)	0.72	2 (6.5%)	2 (6.5%)	1.00
Treatment regimen (n, %)						
Docetaxel	16 (43.2%)	39 (52.7%)		15 (48.4%)	21 (67.7%)	
Docetaxel + trastuzumab	11 (29.7%)	6 (8.1%)		9 (29.0%)	5 (16.1%)	
Docetaxel + trastuzumab + pertuzumab	1 (2.7%)	22 (29.7%)		1 (3.2%)	0 (0%)	
Docetaxel + cyclophosphamide	9 (24.3%)	7 (9.5%)		6 (19.4%)	5 (16.1%)	

Liver dysfunction: grade 1 or higher aspartate aminotransferase, alanine aminotransferase, and total bilirubin levels.

*ECOG* eastern cooperative oncology group, *ER* estrogen receptor, *PR* progesterone receptor, *HER2* human epidermal growth factor receptor 2, *BSA* body surface area, *BMI* body mass index, *CCr* creatinine clearance, *RASIs* renin–angiotensin system inhibitors.

**P < 0.01.

### Evaluation of docetaxel-induced HFS

**Figure 2:**
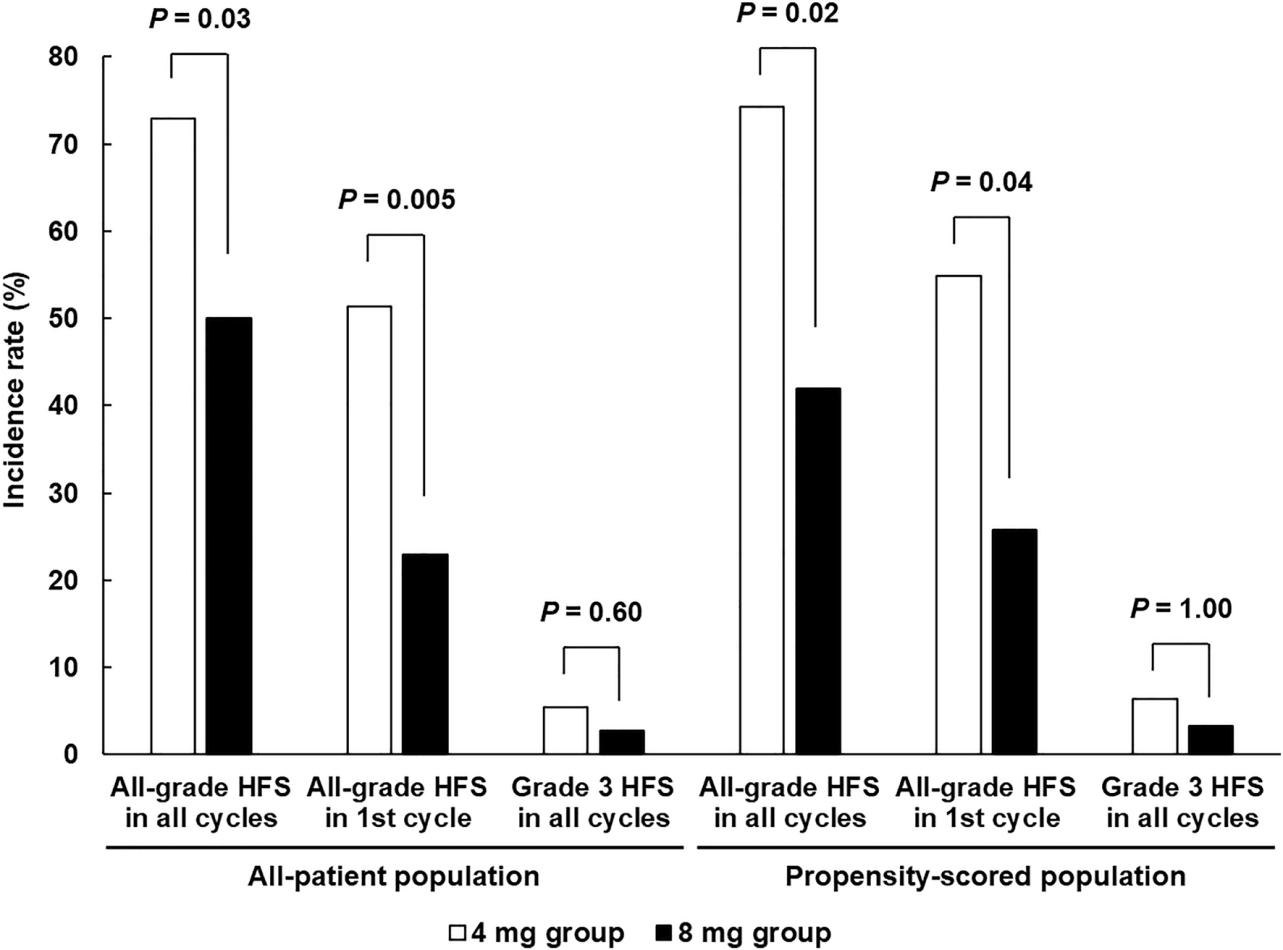
Comparison of docetaxel-induced HFS development in all cycles and first cycle in all- and propensity score-matched populations.

Figure 2 shows the comparison of docetaxel-induced HFS between the 4 and 8 mg groups. The primary endpoint of the present study was the difference in all-grade HFS development during all treatment periods between the two groups in the all-treatment population. Development of HFS was significantly lower in the 8 mg group (50.0%) than in the 4 mg group (73.0%, *P* = 0.03). In addition, its development in the first cycle was also lower in the 8 mg group (23.0% vs. 51.4%, *P* = 0.005). In contrast, the incidence of grade 3 symptoms did not differ between the two groups (2.7% in the 8 mg group and 5.4% in the 4 mg group, *P* = 0.60). These results were also confirmed in a propensity score-matched population.

### Risk factors associated with docetaxel-induced HFS

Multivariate logistic regression analysis was performed to identify independent risk factors for all-grade docetaxel-induced HFS development in all cycles and the first cycle in accordance with previous studies^6–11^. The analysis showed that higher DEX dose was the singular independent preventive factor for docetaxel-induced HFS (adjusted odds ratio 0.35; 95% confidence interval 0.14–0.86, *P* = 0.02 for all cycles; 0.26, 0.11–0.63, *P* = 0.003 for the first cycle; Table 2).

**Table 2:** Univariate and multivariate logistic regression analyses of risk factors associated with the incidence of all-grade docetaxel-induced HFS in (A) all cycles and (B) first cycle in all-patient population.

	Univariate analysis		Multivariate analysis	
	Odds ratio (95% CI)	*P* value	Odds ratio (95% CI)	*P* value
(A)				
BMI (kg/m^2^)				
≥ 25/ < 25	0.73 (0.33–1.61)	0.44	0.74 (0.31–1.75)	0.49
Hypoalbuminemia				
Present/absent	1.02 (0.48–2.17)	0.96	1.27 (0.56–2.90)	0.57
Complications of DM				
Present/absent	0.53 (0.11–2.48)	0.42	0.68 (0.13–3.53)	0.65
Prior anthracycline administration				
Present/absent	1.84 (0.76–4.46)	0.18	2.11 (0.82–5.43)	0.12
Dexamethasone dosage				
8 mg/4 mg	0.37 (0.16–0.87)	0.02*	0.35 (0.14–0.86)	0.02*
(B)				
BMI (kg/m^2^)				
≥ 25/ < 25	0.94 (0.41–2.18)	0.89	1.06 (0.42–2.69)	0.90
Hypoalbuminemia				
Present/absent	0.92 (0.42–2.04)	0.84	1.12 (0.47–2.70)	0.79
Complications of DM				
Present/absent	0.82 (0.15–4.46)	0.82	1.04 (0.17–6.54)	0.96
Prior anthracycline administration				
Present/absent	1.41 (0.53–3.73)	0.49	1.55 (0.55–4.42)	0.41
Dexamethasone dosage				
8 mg/4 mg	0.28 (0.12–0.66)	0.003**	0.26 (0.11–0.63)	0.003**

*CI* confidence interval, *BMI* body mass index, *DM* diabetes mellitus.

*P < 0.05.

**P < 0.01.

### Incidence of gastrointestinal adverse effects and febrile neutropenia

As a dose increase of DEX can affect gastrointestinal adverse effects and increase susceptibility to infection, we additionally compared the patients’ symptoms (Table 3). No DEX dose-dependent increase in adverse effects was observed. Moreover, no patients experienced vomiting and pneumocystis pneumonia or grade 3/4 severe gastrointestinal adverse effects.

**Table 3:** Gastrointestinal adverse effects and febrile neutropenia by study group.

	All-patient population			Propensity score-matched population		
	4 mg group (n = 37) (%)	8 mg group (n = 74) (%)	*P* value	4 mg group (n = 31) (%)	8 mg group (n = 31) (%)	*P* value
Nausea grade 1/2	32.4	24.3	0.37	32.3	25.8	0.78
Anorexia grade 1/2	27.0	31.1	0.83	25.8	32.3	0.78
Febrile neutropenia grade 3	43.2	27.0	0.09	48.4	22.6	0.06

## Discussion

HFS is a management-requiring adverse effect of docetaxel-containing chemotherapy as this treatment is generally administered in outpatient settings. Urakawa et al. reported that although HFS was not a life-threatening symptom, it had the strongest impact on QOL in patients with chemotherapy-induced skin toxicities^23^. Inflammation is one of the causes of HFS, and we have previously reported that systemic DEX attenuates or prevents adverse inflammatory effects in a dose-dependent manner^21,22,24^. Consequently, we aimed to assess the dose-dependent preventive efficacy of systemic DEX against docetaxel-induced HFS in patients receiving breast cancer treatment in a real-world setting.

High-dose DEX administration significantly prevented the symptoms of HFS, including early development, compared to its lower dose. Additionally, a logistic regression analysis taking the forementioned factors into consideration identified high-dose DEX administration as the singular independent preventive factor for all-grade docetaxel-induced HFS development. The results obtained in the present study revealed that systemic corticosteroid administration prevents chemotherapy-induced HFS in a dose-dependent manner, and were consistent with our previous findings regarding other inflammatory symptoms, suggesting that DEX can reduce adverse inflammatory effects in a dose-dependent manner. We also reported that a higher dose of DEX attenuates docetaxel-induced fluid retention^20^. We contemplate that the appropriate DEX dosage for managing docetaxel-induced adverse effects is 8 mg/day, based on our previous and present results. However, the suitable DEX administration period remains unclear. Corticosteroids induce broad adverse effects such as blood sugar elevation and reduced bone mineral densities, which are problematic in longer treatment periods, insomnia, and increased susceptibility to infection^21^. In our previous studies that evaluated the dose-dependent efficacy of DEX against adverse inflammatory effects, relatively acute symptoms, such as insomnia and infection, did not differ between the groups^20–22^. However, we were unable to assess blood sugar elevation and reduced bone mineral density due to lack of data. Consequently, we should also consider these symptoms, and further evaluations, including steroid-induced adverse effects over longer treatment periods, are needed. Furthermore, since patients with breast cancer are a high-risk population in terms of sex, further evaluation in other malignancies can provide additional comprehension.

Nonsteroidal anti-inflammatory drugs (NSAIDs) are promising prophylactic agents for HFS. A meta-analysis of randomized controlled trials suggested that celecoxib, a cyclooxygenase-2 selective inhibitor, reduced the risk of all-grade or moderate-to-severe HFS caused by capecitabine and sorafenib^12^. In addition, urea cream attenuates HFS by reducing local inflammation, which decreases hyperkeratosis and prevents hyperplasia of epithelial cells^12,25^. Since we excluded patients with NSAID co-administration and did not limit our study to urea cream as a moisturizer, their efficacy in docetaxel-induced HFS is unknown. We can consider the use of these medicines for better management of HFS; however, further evaluation of these prophylaxes is required.

The involvement of medical staff is important for better handling of HFS. Essential HFS management by medical practitioners includes patient education, symptom prevention and amelioration, and dose-intensity control^5^. Several reports have suggested the possibility that engagement by medical team including protocol-based pharmacotherapy management or telephone support can reduce the incidence and severity of HFS^26–28^. Consequently, the efforts of medical staff, in addition to appropriate medications, are required for more effective HFS management.

This study had a few limitations that need consideration. First, it was retrospectively performed at a single institution and included a relatively small patient population. Second, we were not able to fully assess the implementation of HFS prophylaxis using moisturizers. However, we confirmed this at every visit and almost all the patients adhered well to using moisturizers. Third, we assessed HFS by referring to the treatment diary, medical interviews, and inspections on the treatment day. Therefore, severity evaluation in some patients may have been insufficient. Fourth, in this study, docetaxel was administered on day 1 alone, which was significantly different from oral medicines such as capecitabine or multikinase inhibitors, which are administered for a certain consecutive period. Since corticosteroids have broad-ranging adverse effects, prolonged systemic administration of these drugs can be problematic. However, it would be useful to evaluate its preventive efficacy on HFS caused by fluorouracil infusion like mFOLFOX6 or FOLFIRI, which are key treatments for metastatic colorectal cancer^29^. Fifth, we excluded patients who were regularly administered NSAIDs. Combination prophylaxis with DEX and NSAIDs can provide promising results. Finally, the patient backgrounds differed according to age, CCr, serum albumin levels, and concomitant pertuzumab use in the all-patient population. However, the same results were confirmed in a propensity score-matched population. In addition, this study had a sex bias, and females are a high-risk population for HFS development. We believe that an evaluation using a well-balanced population with appropriate patient numbers is desirable. Consequently, our preliminary findings need to be confirmed in future studies.

In conclusion, our study suggests that systemic DEX prevents docetaxel-induced HFS in patients with breast cancer in a dose-dependent manner. Evaluation in other HFS-inducing chemotherapeutic treatments and of appropriate DEX administration methods will provide less problematic chemotherapeutic treatments; therefore, further studies are warranted.

## Methods

### Study population

Female patients with breast cancer who received docetaxel-containing treatment at Hokkaido University Hospital between May 2016 and August 2023 were retrospectively assessed. All enrolled participants met the following baseline registry criteria: (1) age ≥ 20 years, (2) 0–1 ECOG-PS score, (3) sufficient data available from the medical records, and (4) acceptable renal and liver functions for treatment induction. Patients who were regularly treated with corticosteroids or NSAIDs, diagnosed with HFS or problematic skin disease at baseline, previously administered docetaxel, or who underwent cooling of hands and feet from treatment initiation were excluded from this study. Patients were divided into two groups: a 4 mg group, which included patients administered oral DEX 4 mg on days 2–4 between May 2016 and October 2018; and an 8 mg group including patients administered 8 mg DEX orally on days 2–4 between July 2017 and August 2023.

This study was approved by the Ethical Review Board for Life Science and Medical Research of Hokkaido University Hospital (approval number: 023-0241) and was conducted in accordance with the tenets of Declaration of Helsinki and the STROBE statement. The need for informed consent was waived by the Ethical Review Board for Life Science and Medical Research of Hokkaido University Hospital due to the retrospective nature of the study.

### Treatment methods

Docetaxel 75 mg/m^2^ was administered intravenously every 3 weeks. Trastuzumab (8 mg/kg at first administration and 6 mg/kg at subsequent administration every 3 weeks), with or without pertuzumab (840 mg at first administration and 420 mg at subsequent administration every 3 weeks), was co-administered in cases of HER2 overexpressed breast cancer. Intravenous DEX (6.6 mg in 4 mg group or 9.9 mg in 8 mg group) and granisetron (3 mg) were administered in the case of docetaxel + cyclophosphamide 600 mg/m^2^, and 6.6 mg DEX was injected in other docetaxel-containing regimens as premedication in accordance with the national antiemetic guidelines^30^. In addition, oral DEX was administered on days 2–4, as previously described. All patients were strongly recommended to undergo skin care with skin moisturizers at least twice daily. Steroid ointment administration or dose modification of docetaxel was performed at the physician’s discretion.

### Evaluation of HFS

All the required information between May 2016 and September 2023 at Hokkaido University Hospital was extracted from the participants’ medical records. In clinical practice, we evaluate HFS incidence and severity by referring to daily treatment diaries, medical interviews, and inspections at every visit in accordance with the Common Terminology Criteria for Adverse Events version 5.0. The primary endpoint of the present study was development of all-grade HFS during all treatment cycles between the two groups in the all-patient population. Secondary endpoints included the assessment of all-grade HFS development in the first cycle and grade 3 HFS. Additionally, we identified the factors associated with development of all-grade HFS. Moreover, propensity score-matching was performed to adjust for the baseline patient backgrounds between the two groups, and the matched data were additionally assessed to confirm the robustness of the all-patient population analysis.

### Statistical analysis

We hypothesized that the incidence of all-grade HFS during all treatment cycles would be 70% in 4 mg group and 40% in the 8 mg group, based on previous reports^14,21,22,24^ and our clinical experience, with a patient ratio of 1:2. The calculated required sample sizes were 37 patients in the 4 mg group and 74 patients in the 8 mg group.

The differences in baseline patient backgrounds between the 4 and 8 mg groups were compared using the Mann–Whitney *U* test for continuous variables and Fisher’s exact probability test for categorical variables. HFS development was assessed using Fisher’s exact probability test. Logistic regression analyses were conducted to identify the independent risk factor(s) associated with development of all-grade HFS. Potential covariates taken into consideration were higher BMI (≥ 25 kg/m^2^), hypoalbuminemia, and complication of DM at baseline, presence of prior anthracycline treatment, and DEX dosage, based on previous studies^6–11^. Propensity score-matching was performed using the following baseline variables: age, treatment setting, hormone receptor expression, HER2 overexpression, BMI, liver dysfunction, hemoglobin level, serum albumin level, DM complications, prior treatment history, and concomitant administration of pertuzumab, pegfilgrastim, and RASIs considering the findings of previous reports^6–11^. To decrease bias with these potential confounding factors, 1:1 matching (without replacement) between the two groups was conducted using the nearest neighbor method with a 0.20 width caliper of standard deviation of the logit of propensity scores.

All analyses were performed using JMP statistical software (version 16.1; SAS Institute Japan, Tokyo, Japan). *P* value < 0.05 was considered significantly different.

### Ethics approval and consent to participate

All procedures in the present study were performed in accordance with the ethical standards of the institutional and/or national research committee and the 1964 Helsinki Declaration and its later amendments or comparable ethical standards. This study was approved by the Ethical Review Board for Life Science and Medical Research of the Hokkaido University Hospital (approval number: 023-0241). Ethical Review Board for Life Science and Medical Research of Hokkaido University Hospital waived the requirement for formal consent for this type of study.

## Data Availability

The datasets used and/or analyzed in the current study are available from the corresponding author upon reasonable request.
